# Uniqueness and Generalization in Organizational Psychology: Research as a Relational Practice

**DOI:** 10.3389/fpsyg.2021.638240

**Published:** 2021-02-04

**Authors:** Giuseppe Scaratti, Silvia Ivaldi

**Affiliations:** ^1^Department of Psychology, Catholic University of the Sacred Heart, Milano, Italy; ^2^Department of Human and Social Sciences, University of Bergamo, Bergamo, Italy

**Keywords:** situated and relational research, epistemology, ontology, gnoseology, methodology

## Abstract

The paper addresses the epistemological and theoretical assumptions that underpin the concept of Work and Organizational Psychology as idiographic, situated, and transformative social science. Positioning the connection between uniqueness and generalization inside the debate around organization studies as applied approaches, the contribution highlights the ontological, gnoseological, and methodological implications at stake. The use of practical instead of scientific rationality is explored, through the perspective of a hermeneutic lens, underlining the main features connected to the adoption of an epistemology of practice. Specifically, the contribution depicts the configuration of the applied research as a relational practice, embedded in the unfolding process of generating knowledge dealing with concrete social contexts and particular social objects. The discussion of a case study regarding a field research project allows one to point out challenges and constraints connected to the enactment of the research process as a social accomplishment.

## Introduction: Producing Relevant Knowledge In/For Organizations Through Work and Organizational Psychology

In 2011, Bartunek published in the *British Journal of Management* (*BJM*) a paper, whose title (“What has happened to Mode 2?”) questioned the extent of the diffusion and impact of Mode 2, a research approach proposed by Gibbons (Gibbons et al., 1994) for bridging the relevant gap between academic knowledge and practical knowledge, improving ways of knowledge production to link together rigor and relevance, research and action, and theory and practice. Bartunek advocated for “the restructuring of academic institutions to allow better knowledge exchange and distribution consistent with Mode 2 approaches, the creation of new measures of academic achievement, the fostering of executive doctoral programs and the creation of journals and associations that would foster trans-disciplinary research that responds to applied problems” (Bartunek, 2011, p. 556).

As far as abductive could be the reference to the reviewing practices in use among journals with high reputation (in terms of IF), Bartunek’s questions and suggestions seem not to have received satisfactory answers if we look at the difficulty of publishing articles that conceive WOP as an idiographic, situated, and transformative social science.

Just to refer to diffused examples detectable by the articulated field of sentences and comments coming from reviewers that circulate among scholars through their narrative exchanges, we can observe that one should read Orlikowski (2000), acknowledge the related theoretical background, and know her concept *of using technology through a practice lens* before asking about the meaning of the word “*use*.” Or it could be asked why a professional report or a journal for practitioners should be inevitably categorized as *pseudoscientific*, preferring papers published in “A journals” and not on doing research that influences practice (Lawler, 2007); further, other good thoughtful reflections should be triggered by reading the chapter of Shotter and Tsoukas (2011) related to *theory as therapy* inside the theorizing in organization and management issues.

Whatever the considerations relating to the consistency of such comments and the necessary humility expected by each author in accepting criticisms and suggestions to improve a submitted work, at stake here are implicit and taken-for-granted concepts about understanding research, science, and the relationship between scientific knowledge and practical knowledge – even more so as the reference of the special issue is not to psychology in general but to the specific field of Work and Organizational Psychology (WOP).

The paper addresses the epistemological and theoretical assumptions associated with the need to go beyond the linearity of theory application, moving from the content of knowledge to the locally acknowledged use(s) of knowledge. We position the matching between uniqueness and generalization inside the debate around *organization studies as applied science* and the sought connection between theory and practice.

The aim of the paper is to support a research perspective oriented at producing relevant knowledge by working *with* – and not *on* – people (Shotter, 2010) and by confronting with problems that cross the lives of real organizations.

Our purpose in this article is to explore the following questions:

- Which theoretical frameworks and conceptual anchors underpin the representation of WOP as idiographic, applied, and situated science?
- Which ontological, gnoseological, and methodological implications can be highlighted stemming from the assumption of the epistemic significance of the particular?

To answer the research questions, we address and develop the contributions regarding the adoption of *practical instead of scientific rationality* (Tsoukas, 2009; Sandberg and Tsoukas, 2011; Shotter and Tsoukas, 2014), seeking to bridge the gap between theory and practice that hinders applied social sciences. We argue that the tension of generalizability, in the specific field of WOP, concerns the construction of knowledge within and among organizations that supports the acquisition of a more “pragmatic science” (Anderson et al., 2001) and relevant knowledge (Jarzablowski et al., 2010) embracing academic-practitioner collaboration (Bartunek, 2007) and going beyond a narrowly circumscribed areas of study, achieving box-breaking research (Alvesson and Sandberg, 2014).

The paper unfolds as follows. We begin by exploring the epistemology of WOP as an idiographic and applied science, addressing the socially constructed nature of the phenomena under consideration. In this perspective, uniqueness and generalization deal with research issues generated by problems of real organizational action, negotiated in social and tangible scenarios that contribute to determining the objects of study (Bartunek et al., 2006; Pfeffer, 2007). Thereafter, we point out the most relevant implications addressing the ontological, gnoseological, methodological, and practical dimensions at stake. We then discuss a case study related to an on-the-field research project, highlighting the relational aspects embedded in the unfolding process of generating knowledge within a concrete social context, the actors involved and their representation of the object (whose demarcation is not immediately obvious), and the ways to access local and situated knowledge connected to the experience of people involved. Finally, we conclude by synthesizing the elements that allow defining such type of research as *situated*, *transformative*, and *relational*, suggesting hints for further research.

## Theoretical Framework: Toward an Epistemology of Particular and Practical Rationality

### WOP as Idiographic and Applied Science

As an *applied science*, WOP is routed in a postmodern trajectory (Kvale, 1992; Gergen, 1994, 1995) and overcomes a prescriptive and routinized approach to conduct research, having been revised (or even upset) through the last decades of debates about the linguistic turn (Wittgenstein, 1958), the narrative turn (Bruner, 1986, 1990; Sarbin, 1986; Polkingorne, 1988), the practice turn (Schatzki et al., 2001; Reckwitz, 2002; Whittington, 2011), the reflexive turn (Schön, 1991; Macbeth, 2001; Cunliffe, 2002, 2003; Cunliffe and Easterby-Smith, 2004; Scaratti and Ripamonti, 2015), the interpretative turn (Hiley et al., 1991; Reckwitz, 2002), and the socio-material turn (Dameron et al., 2015). A different perspective of research emerges, one that can help people engaged in actual organizational contexts cope with processes of learning on the fields, connecting action and thought and trying to open new visions not yet available for transforming and improving their daily practices. Such an approach is capable of relaunching and promoting the genuinely practical and applied hearth of the discipline, as required to answer to the challenges produced by the crisis in complex and unstable organizational contexts. Being inspired by a dialogical and transformative perspective (Cunliffe and Scaratti, 2017), we call for a *relational research approach* in the WOP field, highlighting both the epistemological assumptions and the methodological options that are consistent to such perspective, as well as the issues concerning the validity and common criteria for good practice in such relational and transformational process-oriented research. We draw on a hermeneutic lens (Bernstein, 1983; Dreyfus, 1991; Smith, 1997) that moves from phenomenology through social representations to semiotic, focusing on knowledge as structurally connected to the experiences lived by subjects, their contexts of reference (*lebenswelt*) and the meanings they share in them. Hence, there is a need to legitimize the existence of a plurality of scientific paradigms, as it is possible to formulate knowledge in distinct ways and, therefore, to attribute certain properties and relationships to the universe of objects considered. The different paradigms can be conceived and declined in dialogue, acknowledging a monism of principles (plausibility of the assertions/intersubjectivity; tension for generalizability/transferability) and a multiplicity of applications and procedures of empirical documentation imposed by the specificity of the contexts (Cardano, 2001).

Thinking of WOP as an idiographic and applied science entails the assumption of an epistemological position, seeking for the validity of a situated approach in a specific field of psychological research and intervention. We claim the concept of validity to be intertwined with social validation and acknowledgement: A research or intervention process is valid if its relevance is recognized by the actors that collaborated to produce knowledge and if it is coherent with the conventional rules shared inside a scientific community. At stake, since the focus of the special issue is about critical perspectives on replicability in WOP research, are the uniqueness of idiographic studies in this specific field, their validity beyond the boundary of a specific case, and how and in which sense particular findings coming from an ethnographical design or case study can be generalized.

Accordingly, we follow the contribution of Tsoukas (1989, 2009) who, drawing on Toulmin’s plea for the need to overcome a decontextualized lens looking forward to an ecological style of research (Toulmin, 1990), affirms the epistemological importance of what is particular and local.

Tsoukas addresses the *epistemic significance of the particular*: It relies on the possibility to enrich the general concepts related to the issue under study since the situational uniqueness of specific phenomena provides analytical refinement of what is currently known. As highlighted by Wittgenstein (1958), the commonality among similar phenomena does not refer to a specific analytical definition or single features but depends on family resemblances connected to the possible multiple usages of their related concepts. Hence, the relationship between general and particular touches upon a heuristic generalization that allows a better understanding of the phenomena at hand, putting in dialogue thick descriptions and available accounts (in their uniqueness and idiosyncrasy) with theoretical frameworks at stake. In an unfolding analytical/theoretical accomplishing process of generalization, the particularity of a singular case provides several possible interpretations, while general concepts take a distinct form in specific settings historically shaped and dynamically reshaped, due to the *radial structure* of concepts (Tsoukas, 2009, p. 290). The potential of generating knowledge from idiosyncratic reality is embedded in the unicity of each case study, since its situated original configuration provides a *portrait of the world* (Shotter and Tsoukas, 2011; Scaratti, 2014), allowing the researcher to highlight different repertoires (perhaps new but anyway interesting ones). For this reason, Tsoukas (2009, p. 297) upholds that what happens in particular cases does matter for theorizing as an analogical process, to enhance the understanding of puzzling phenomena. The deepness, density, and thickness of the particular, detected and depicted in its situated configuration, open both vertical (connection with many different conceptual interpretations of the phenomena) and horizontal extensions (plural ethnographical accounts and perspectives through which the phenomena can be seen). The answer to the question “what is the case of” or “what is going on here” does not refer to a specific theoretical issue or concept but rather to the intertwined tangle represented by the object of study. This object can be conceived as a runaway object (Engeström, 2008), progressively defined, shaped, and shifting, coming from the unfolding achievement of an accord between the knowledge interests of researchers (oriented through theoretical background), the needs of the practitioners, and the relationship among co-researchers (who are involved and with what expectations) in the common game for generating relevant knowledge (Scaratti and Ivaldi, 2015).

Due to this social configuration (Scaratti et al., 2017a) of the objects of inquiry, in the field of WOP, themes and issues become *social objects*: as Garfinkel (1947) claimed, they are characterized by “unrepeatable uniqueness.” Compared to “specific referent symbols” (related to defined classification and determination of boundaries, as in the traditional way of conceiving science), they are rather “expressive symbols,” with a *requiredness* that refers to semantic and moral values, linked to their context of use regarding the meaning they assume. As social objects, they reveal an *indexicality*, which, in semiotics and linguistics, refers to expressions that are comprehensible in the concrete context in which they are spoken and used (Gherardi, 2009). Social objects convey a socio-material dimension that links discourse to social practices, as the result of interactions that take place in daily organizational and personal life, through the connection of material and immaterial elements (Suchman, 1987; Lave, 1988). Those interactions give rise to an *order of life* that shapes and recursively re-produces the interconnected, shapeshifting activities and practices that connect individual, collective, organizational, and institutional realms through different kinds of intermediaries (tangible objects, artifacts, people, speeches, texts, etc.). Studying social objects, made of a web of tangible and symbolic dimensions, the researcher faces critical incidents, turning points, dynamic evolutions, skills in specific situations, and practices in use taken for granted, employed to make a sense of one’s world (Ivaldi et al., 2015; Ivaldi and Scaratti, 2016, 2020).

### Achieving a Logic of Practice

Sandberg and Tsoukas (2011) develop the above orientation arguing for the acquisition of *a logic of practice instead of scientific rationality* to bridge the gap between theory and practice in the applied social sciences. They provide an onto-epistemological premise upsetting the traditional rationality in use in managerial and organizational theories that hampers the possibility to reduce the distance between scientific knowledge and managerial practice. Since organizational practices are constituted and enacted by actors, we need to detect the logic of practice, being close to the way actors enact their practices, going beyond a representational view of theory, based on the rational subject-object relation, depicted by Shotter and Tsoukas (2011) as unrealistic and misleading.

The principal limit of *scientific rationality* is its context-free assumption, related to the need to avoid biased, non-rational, and subjective knowledge compared with the exact and rational canons of the scientific method, focused on a well specific object, defined by a theory and studied through an experimental laboratory approach (being recursively replicable), exclusively achieving empirical evidence. Such a disconnection of knowledge from its social contexts impedes the researcher from grasping the whole meaning of the activity practitioners deal with, the situated uniqueness of their contextual practice, and the multiple ways through which they experience time.

At the opposite, the *logic of practice* detects and grasps the entwinement of ourselves, others, and things in a relational whole: At stake is the acknowledgement of the socially constructed nature of the phenomena under consideration, since knowledge emerges from the never-ending transactions and negotiations through which subjects shape and give sense to their systems of activity. As proposed by an idiographic perspective, practical rationality considers activities as contextualized, characterized by the indexicality of structure through which people make their ordinary practices accountable, organizing their everyday experience. Hence, it is impossible to avoid everyday life situated contexts as a primary source from which humans derive their meanings and the way they act. Researchers must deal with the silent organization (Romano, 2006) as the symbolic and cultural terrain that directs action and work practices, where the situated and implicit knowledge is located. This framework is exposed to the constant interweaving of social negotiation and attribution of meaning to the complexity of reality, a condition for, and simultaneously the results of, relational dynamics and exchanges.

The logic of practice entails conceiving knowledge as particularly emerging from the ceaseless transactions and negotiations through which subjects shape and give sense to their systems of activity. Such approach also highlights the need to address tacit and taken-for-granted knowledge that is embedded in daily practices and routines: The focus of attention, therefore, moves from the content of knowledge to the locally legitimized uses of knowledge (Gherardi, 2019). Knowledge production requires the unavoidable involvement of the actors/authors/researchers in the recursive and constant interpretation of the polyvocality or referential accounts, enhancing social exchanges and reasoning that mobilize the interpretative models of the world and the network of symbolic meanings to which people refer in their organizational and workplace experience.

The perspective detailed above rests on a consistent debate that has accompanied the transition toward a post-modern conception of the human sciences. As Latour (2018) evokes, with the thought-provoking metaphor of landing (getting close to the earth), we need to overcome an exclusive *gravitational* epistemology of movement, achieving a multiparadigmatic view when we are studying social life with its intertwined multiple movements (birth, death, generation, collaboration, conflict, singular, collective, material, and immaterial developmental dynamics).

Since the intention to deepen the plural and articulated epistemological debate of the past decades cannot be extensively covered in this paper, we want to at least address the contribution of Reckwitz (2002) who encompasses the main paradigmatic turning points analyzing the relationship among different social and cultural theories, seeking for a theory of social practices. Reckwitz’s contribution sheds light on the historical emergence of the logic of practice as a result of the culturalist revolutions in the 20th-century philosophical and epistemological debate, running from structuralism and semiotics through phenomenology and hermeneutics, till the Wittgensteinian language game philosophy, acknowledging “the implicit, tacit, or unconscious layer of knowledge which enables a symbolic organization of reality” (Reckwitz, 2002, p. 246). Reckwitz’s analysis provides both a complex view of the field of cultural theories and a distinctive position among them of the practice theory that localizes the social dimension not in mental qualities, neither in discourse nor in interaction, but in practice as “a routinized type of behavior which consists of several elements, interconnected to one other” (Ib., p. 249).

Compared to traditional views (the *homo economicus* perspective in which action is the result of individual/rational interests; the *homo sociologicus* lens in which action is the result of complying with collective norms and values), the cultural theories conceive human beings as agents who interpret the world (body, mind, things, knowledge, discourses, language, structures and processes, and agency) according to forms embedded in collective cognitive and symbolic structures, in a “shared knowledge” that enables a socially shared way of ascribing meaning to the world (Table 1).

**Table 1:** Cultural and practice theories turn.

	Mentalism	Textualism	Intersubjectivism	Practice theory
Locus of the social dimension	Mind	Discourses	Interactions	Practices
Body	Inside-outside distinction between mind and body An epiphenomenon (mind/brain debate)	An object of cultural meanings	“Bodily” basis of action A referent of propositions	Learn to be bodies in a certain way Regular, skillful “performance” of (human) bodies
Mind	An ontological realm of the “inner” which is distinct from outward behavior	A cultural ascription carried out in certain types of discourses	The mental appears as a socialized result of social rules and meanings from outside to inside	Sets of mental activities Routinized ways of understanding the world, of desiring something
Things	Objects of knowledge and thus symbolic categories	Meaningful entities	Objects of the knowing subject	Resources for action Components of social practices mold social practices
Knowledge	Understanding-enabling character of knowledge	Knowledge as a code	The background of understanding on the part of the agent	Ways of understanding, wanting, feeling, and knowing how largely implicit and largely historically culturally specific collective shared knowledge
Discourse/language	A mental set of competences	Discourses as extra-mental and extra-bodily patterns	Speech acts	Language exists only in its (routinized) use
Structure/process	Oversubjective mental categories	Autopoiesis of codes in a sequence of discursive events	“Consensus” of meanings	Practices as routines Reproduction of social order
Agent	Consciousness of mind	A discursive subject-position	“Speakers” who interact with one another	Carrier of social practice

Inside the phenomenological, hermeneutic, linguistic, and interpretative turn explored by Reckwitz, the practice theory provides a novel picture of social and human agency, among different cultural theories, focusing on the conditions of human action and social order.

The focus is on the implicit, tacit, or unconscious layer of knowledge that enables a symbolic organization of reality, dealing with the nexus of doing and saying, since practice is a “type” of behaving and understanding that appears at different layers and at different points of time, carried out by different material and immaterial dimensions that give meaning to the world in a contingent way.

### The Transformative and Social Accomplishment Stance of Applied Science

Sannino (2011) points out another useful approach to differently conceive social science and the transformative nature of research, addressing the principle of ascending from the abstract to the concrete as the fundamental method of dialectical thinking, explored and developed by the activity theory approach (Engeström, 2015). There are two types of abstractions: empirical and theoretical. Empirical abstraction is a classification of superficial features of phenomena. Theoretical abstraction concerns the identification of the genetic origins of phenomena that may externally be not alike at all. A theoretical abstraction is based on a functional relationship, also called a germ cell. While observation and categorization are actions at the root of empirical abstraction, practical transformation, change, and experimentation are actions at the root of theoretical abstraction. Theoretical abstraction allows one to generate and project complex, theoretically mastered, concrete manifestations and developmental forms of the reality under scrutiny. The example of axes and saws is emblematic in this regard: while an empirical abstraction seeks for common features and aspects of the different things, connecting them to a concept at hand, the theoretical abstraction detects a sensory objective activity (they are both tools used in the wood processing) that conveys possible relationship and transformations. In Davydov’s (1984) words: “The substantially general is a particular relation of real objects which … does not exist outside development and transformation” (p. 23) and “theoretical thinking is based on sensory objective activity which reconstructs and transforms the world around us” (p. 25).

We can connect this statement with what Kurt Lewin said about the possibility to know something only through trying to change it. Changing practices and transforming activities require dealing with critical events, thus identifying the embedded problem or challenge stemming from daily experience and activity in order to detect and to focus on the emergent early symptoms (Engeström and Scaratti, 2016; Ivaldi and Scaratti, 2016).

Hence, we need to intercept contents that are immersed and submerged in the practices and to approach and question, together with people, an everyday life studded with contradictions to cope with and opportunities of change development to be improved, detecting and transforming them through processes of expansive learning (Engeström, 2015).

Developing the issue of the fourth generation of activity theory (Engeström and Sannino, 2020; Sannino, 2020) that conceives heterogeneous work coalitions aimed at resolving wicked societal problems, dealing with runaway objects and creating sustainable and equitable alternatives to the mainstream approach, Engeström (forthcoming) calls for a concept formation *in the wild*, exploring the collective creation and use of concepts in everyday activities. The metaphor points out how human actions and cognition are never fully predictable or programmable since historical, social, cultural, relational, discursive, practice, material, and immaterial conditions influence their accomplishment and impede their total control. Every concept is grounded in embodied action and artifact-mediated enactment in the material world. The study of collective creation and use of concepts in everyday activities opens an innovative field of inquiry and investigation, detecting how people engaged in actual organizational contexts are asked to explore processes of learning from the fields, connecting action and thought and trying to open new visions not yet available for transforming and improving their daily practices.

The wild configuration of concepts entails the possibility to look at idiosyncratic data as the manifestation of the inexhaustible knowledge embedded in the concrete reality, revising and redrawing boundaries in use.

Similarly, Feldman and Orlikowski (2011) underline the idea of scientific *research as* an *unfolding process of social accomplishment*, discussing the theoretical approach to practice that underpins the relevance of the particular for a proper configuration of an applied research. The *practice lens* opens new and decisive perspective in the study of organizational phenomena, detecting the multifaceted, complex, and transient configuration of the phenomena at stake. They are characterized by three constitutive features: dynamic (due to the emerging and improvised forms of the activities under study), relational (related to the reciprocal and mutual constitution of different elements, humans and not humans), and enacted (knowledge is grounded in everyday practice and refers to an ongoing accomplishment achieved through actions interpretations and material and immaterial agency).

Since the development of the organizational and managerial phenomena occurs through the enactment of them, we have to get close to the unfolding accomplishment of these processes. It is in the situated use in specific contexts of social practice that things acquire their meaning. The usage is both bounded and open-ended, and for this reason, dealing with particular cases provides a better understanding of the phenomenon at hand: the particular enriches the general.

### The Particular as a Mirror of the Whole

Engeström (2020) points out the relation the particular has to the whole, exploring Wagenschein’s *exemplary learning* and Meyer and Land’s *threshold concepts* (Wagenschein, 1968; Meyer and Land, 2005): both are akin to a portal enabling a new and not yet explored way of thinking about something, as transformed forms of understanding, interpreting, or viewing something, overcoming the dualistic separation of everyday and scientific concepts.

Following that, it is possible (and needed) to overcome an only statistical view of generalization: Seeking similarities among phenomena and objects is not a matter of clear boundaries among common properties but rather it refers to a wide variety of ways in grasping family resemblances. Like Geertz (1973) recalls, the statistical inference is not the only one that can guarantee the passage from local truths to general visions.

Each case conveys a contribution that specifies general concepts and accumulates knowledge beyond any epistemological dualism, seeking for relevant dualities.

Indeed, assuming an epistemology of the particular as well as practical rationality entails a specific conception of social science as applied science, acknowledging that this perspective can enrich the possibilities of our relationship and engagement with the world.

Of course, if the need is to find a vaccine that contrasts the COVID-19 pandemic, we must set up an experimental science capable of arriving at a reliable and safe solution, based on randomized control trials and laboratory’s studies. In this case, the adoption of a subject-object epistemic stance, by which the scientific community confirms the validity of a new scientific discovery repeating the research that produced it (see the National Academies of Sciences, Engineering, and Medicine “Reproducibility and Replicability in Science”^1^), is proper and suitable, together with the connected implication of statistical reproducibility and replicability.

On the other hand, if the request is to understand how to manage health practitioners during an emergency related to a pandemic event, we have to adopt an epistemic stance oriented to the particular context and practical rationality, providing accounts of organizational practice as enacted and seeking for relevant, actionable, rigorous albeit situated knowledge, that can help people in their challenging practice. The validity of a situated dialogic transformative research, in this sense, cannot be ensured by the research rigor by itself. Nonetheless, rigor is necessary to produce knowledge in the form of *repertoires of actions*. Repertoires of actions can be translated and transported into other contexts: We state that the only possible generalization concerns the “ways of functioning” of social contexts in some specific situations.

At the same time, such accounts enhance what we know about managing the unexpected (Weick and Sutcliffe, 2001), assuming different and more heuristic generalization of the acquired knowledge, its uniqueness being historically and contextually grounded. The possibility of a connection between research and action implies a constant regulation between reflective distance and immersion in contexts, overcoming an idea of rigor that can only be traced back to criteria of linear causality, correlation rules, and forecast control (difficult to do if applied to the study of organizations and human systems). Rather, we are required to achieve more awareness and congruence of interpretations for a consensually validated understanding, according to a concept of generalization not in terms of mechanical repetition, but as a re-proposition in other contexts of the repertoires acquired and consolidated by a critical reflection.

At stake is the endeavor for a theory of technique able to combine the problem/issue of the research study, the suitable conceptual framework for it, and the adoption of proper and coherent methods and tools to gather the empirical data related to the defined research questions (Bosio et al., 2012).

For an applied social science as WOP, this entails that relevant knowledge does not emerge exclusively from one-dimensional inquiry process: situated meaningful knowledge is produced also (and often above all) by addressing the multivocality of organizational contexts, namely, by listening to the multiple and often competing discourses and interests that cross the organizations.

Conceiving WOP as an applied science conveys meaning related to coping with different fields, facing situations of imbalance and transformation, scenarios of regression and uncertainty, processes of rapid organizational change, and new solicitations to personal/professional identity deriving from an evolving world of work and organizations. The assumption of practical rationality becomes decisive by virtue of the situated and negotiated generation of the meanings that the subjects exchange in social situations, seeking for the achievement of critical thought oriented toward action and reflection, suitable to cross and creatively open experiences, fields of action, contradictions, and diseases, being able to jump into the unknown and shape different and innovative practices.

The relationship between the applicative stance (WOP as an applied science) and the singularity (uniqueness or particular) rests on the concrete and situated experiences of the subjects as the constitutive and privileged ground for the production and reproduction of knowledge. WOP is applied as “sticky,” clinging to working, knowing, and organizing practices, to the implicit forms with which the actors carry out the activities in their contexts, grasping the ways through which they shape and reshape their own cognitive and experiential field.

To summarize, the applicative stance is required because

- the reality we face up to in terms of research and production of knowledge is the result of a process of social construction;
- the issues WOP deals with manifest themselves more as needs than as defined problems, becoming specific demands as a possible (not taken for granted result) process of social negotiation, neither predictable nor guaranteed to be successful;
- the knowledge produced is situated, more precisely built into specific situations and contexts and relationally and processual in its framing, as an outcome of exchanges, transactions, and negotiations between researchers and subjects.

At stake for WOP as an applied social science is the need to go beyond a procedural and formulaic approach to the object under study (Alvesson and Gabriel, 2013), anchoring the construction of knowledge in situations, to enhance action at various levels and promote a reflexive stance (Cunliffe, 2002, 2003). Such a perspective looks at the organizational contexts as practiced places of professional biography and help subjects to recompose, in terms of meaning, a conversational dialogue with one’s work and knowledge related to the action (Cunliffe and Scaratti, 2017). The applied configuration of WOP emphasizes the need to overcome the traditional rationality that sees the particular as a pretext to apply the explanatory properties of a theory, following a temporal logic that places theory first and then its implementation. Rather, this approach seeks to pursue a reversed outlook, assigning a temporal priority to the field of action that suggests possible trajectories for the development of a theory.

### The Evidence Challenge

Strictly connected to the applied dimension of WOP is the issue of the evidence-based foundation of each social science, seeking for a kernel of truth as a proper tension toward the purpose of matching rigor, relevance, and empirical support. The orientation of evidence-based management (EBM) is moreover now consolidated in the literature of our scientific community (Pfeffer and Sutton, 2006; Lawler, 2007; Rousseau and McCarthy, 2007; Rousseau, 2012; Barends et al., 2014). The opportunity and need to refer to practices supported by evidence in the managerial and organizational processes seems to be an unavoidable and completely obvious position. Indeed, who would renounce using the most adequate scientific information available in everyday life if it can improve the knowledge, judgment, and skills of those who work? It would be like defending choices that are not based on a reasoned weighting but supported by superficial readings, by bizarre and eccentric positions, or by erroneous and counterproductive assumptions. The rhetorical force of these statements is indisputable, so in a certain sense, we cannot help but call ourselves evidence-based oriented.

Reflexively dialoguing with this orientation, Hammersley (2013) points out the risk of an erroneous attribution of superiority to specific methods and research results, attributable to randomized controlled or equivalent studies. Such an attribution triggers the expectation of a linear transposition from results to practice, understood as the direct and temporally sequential application of the research findings thus acquired to the practice. The issue concerns, on the one hand, the actual and not only declared legitimacy of the various sources from which to draw the evidence and, on the other hand, the role assigned to the expert judgment in the translation of actionable knowledge into practice.

Concerning the first aspect, it is possible to observe how in the most up-to-date approaches to EBM (Barends et al., 2014) explicit reference is made to four forms of evidence to draw upon for the exercise of a reasoned, explicit, and judicious decision-making process: research results; data, events, and factual elements collected by organizations; the professional experience and judgment of practitioners; and the values and interests of stakeholders. It is an important reconfiguration of evidence-based guidance, adapting to recurring situations in which specific research results are not available (or those accessible are unsatisfactory), as well as to contexts and scenarios where taking immediate decisions under conditions of high uncertainty is a normal rather than exceptional condition (Weick and Sutcliffe, 2001).

The second aspect concerns the use of experiential knowledge, capable of reflection and critical thinking, interpretative discernment, and expert judgment. Such judgment certainly makes use of different sources of evidence available, avoiding assigning rankings of greater/lesser validity to one or the other, modulating them with reference to local needs, to be discerned and evaluated. Hammersley emphasizes the enhancement of tacit judgments, local knowledge, and contextual skills as relevant elements that can generate proper and suitable situated interpretations. Evidence is not seen as a result of abstract procedures or standardization techniques but of careful consideration of both the questions to be answered and the context variables that determine the material and social conditions of the research. The access to evidence requires both the configuration of cognitive needs to be met (in a declination of theory-driven and problem-driven approaches) and the analysis of context variables and interpretations of the field (according to a context-driven declination of research). Hammersley quotes Polanyi (1962), who advocates the relevance of tacit and personal knowledge, referring to the theme of *phronesis* as a form of practical knowledge (or wisdom), that practitioners use to produce informed judgments. It is thus possible to combine it with the *episteme* as two legitimated and relevant forms of knowledge, capable of orienting practical actions within the multiple human contexts in which the subjects are involved. While episteme provides analytical rationality, phronesis conveys hints and adaptive suggestions for tackling with uncertain and unexpected situations and facing the particularity and contingency of contexts.

A possible objection to the above thoughts could be the consideration that orienting research to the generation of useful and expendable knowledge for the practitioners involved is equivalent to an operation of mere subservience to opportunistic and instrumental business needs. In this regard, Schütz (1970) proposes a distinction between *imposed relevance* and *intrinsic relevance*, the latter relating to forms of research driven by amazement, surprise, and curiosity with respect to objects of investment and inquiry interests. However, while it is worthy (and ethically requested) to underline the risk of instrumental enslavement of applied research, it is quite another thing to deny the possibility (and the necessity, in some cases) of forms of research oriented to knowledge questions emerging from the concrete organizational scenarios. Such form of research is generated by the work experiences of the subjects, with the purpose to activate their elaboration and possible suitable evolution. The generation of scientific problems arises from the sociocultural contexts in which research is immersed and what to give relevance varies according to different and plural interests, positions, and perspectives. The involvement of stakeholders becomes an essential condition for identifying relevant needs and sustainable forms of investigation, related to the configuration of problems for their understanding and transformation.

Such a relationship with practice does not constitute a condition of subservience of the research but outlines a relevance “imposed” by the contexts (as it is negotiated and socially constructed), which becomes of intrinsic relevance.

A construct of evidence is emerging as *practice-based evidence*, as a social process of knowledge production, connected to the production of knowledge fed both by *a research-based practice* and *practice-based research*.

The acquisition of empirical evidence in the context of WOP relates to the need to define and agree on “good” research questions (for example, the exploration of how organizational actors interpret and manage operational practices or systems of activities). The achievement of such goodness requires the availability of different expertise and knowledge to be recognized and legitimized. On the one hand, making practical and situated knowledge absolute means preventing its critical processing and ignoring its possible fallacy (some practical knowledge is dysfunctional and incongruous with respect to the work objectives pursued and, consequently, must be unlearned). On the other hand, conceiving scientific knowledge as absolute and primary feeds the late and neo-positivist illusion of an objective gaze on the world. This hinders the possibility to grasp the conventional, practical, and linguistic conditions of the situated and local dimensions, thereby determining its own irrelevance.

## Ontological, Gnoseological, Methodological Implications of a Relational Practice Research

In the previous paragraph, we have underlined the possibility of considering not only traditional ways of producing knowledge, according to theoretical-disciplinary logics that guide the identification of research problems and mark linear sequences of application of the hypotheses and verification of results. We claimed the opportunity also of processes of social production of knowledge, in which problems are generated within contexts, through explicit and implicit forms of negotiation and social construction that involve multiple actors, different disciplinary knowledge, and material and immaterial dimensions. The possibility to produce relevant, cumulative, and significant knowledge with (not on) organizations and people implies the need to start from the configuration of questions generated by real problems. It also requires distinguishing the typical approach of natural sciences from that of the human and social sciences, overcoming a traditional idea of generalizability, without renouncing to a broader and deeper understanding, limiting the risk of false conclusions.

Assuming an epistemic awareness of a science immersed in the world and underpinned by an epistemology of the particular and practical rationality, it is possible to question a unitary vision of modern science by adopting sensitive and appropriate ontology, gnoseology, and methodology, to grasp the symbolic dimensions present in the phenomena studied and related to the meanings people attribute to their work and organizational experience.

### The Ontological Option of Mediated Realism

Our orientation stems from a phenomenological-hermeneutic perspective based on an *ontology of critical and mediated realism* (Porter, 1995; Altheide and Johnson, 1998; Mantovani, 2000). It is connected to the use of material and immaterial intermediaries (pre-understandings, devices, and artifacts), related to a socio-constructionist paradigm and a logic of affordance through which material things convey cultural implications and potential meanings. Such ontological configuration leads to the connection of a cultural vision of semiotic processes (for which our modes of signification and referencing would take place according to a process of social construction and negotiation, mediated by artifacts and material/immaterial conditions), with a position of moderate realism. This stance is linked to the limits of interpretation as a condition of hermeneutic activity (Eco, 1997): the games of interpretation of facts and situations are recognized as plural and multifaceted but not infinite, since not all interpretations are accepted as appropriate, adequate, and equal. The reference to critical realism means that it is the belonging to social contexts of participation and communication (Wenger, 1998), connected to the concrete practices in which people are involved (Suchman, 1987), that configures reality. Subjects attribute common interpretations to what happens in their experience through concrete processes of connection between cognition, culturally mediated action as well as interaction (Alby and Zucchermaglio, 2006).

The *mediated realism* solicits the plurality of applications and procedures for the construction of empirical documentation and advocates for conditions of situativity of the objects of study. This interpretation is consistent with the invoked need to move along a variety of positions, perspectives, and interpretations that can be activated by drawing the relationship between worldviews and ways of theorizing, seeking a common basis of understanding, according to knowledge demands pursued. One must clarify the goals and knowledge questions for the analysis of organizational contexts to be increasingly acknowledged and relevant.

### The Gnoseological Assumption of Sticky-to-Practice Knowledge

From a *gnoseological point of view*, such an ontology entails a specific concept of knowledge as situated and context-driven. We position ourselves within a constructionist theory of knowledge that underlines its situated, contextual, and distributed dimension (see Goodman, 1978; Schön, 1983, 1987; Scribner, 1986; Brown and Duguid, 1991; Tsoukas, 1996; Schatzki et al., 2001; Orlikowski, 2002), together with the multiple ways of knowing in use and the consequent need not to separate cognitive products from the socio-cultural conditions of their production. Contextual knowledge is predominantly a situated knowledge, as it is connected to a corporeality, a sensoriality, and an esthetic (perceiving and sniffing things; grabbing something on the fly; sensing; straining the ear; having an eye and nose; …). It is generated through interactions and social relationships through which the game of attributing meaning to events unfolds. Such knowledge is expressed through linguistic, discursive, and conversational practices, understood as systems of action, and it is anchored to material and physical components that characterize the workplace situation.

The emphasis on the construct of *knowing in practice* (Lave and Wenger, 1991; Gherardi, 2006) and knowledge as *sticky to practice* (Brown and Duguid, 1991) highlights the need to consider the dimensions of tacit knowledge and the methods of its production and circulation. We think about knowledge as located within defined operational contexts, about practice as deposited (tacit, implicit, and incorporated) in the shares and routines; tacit knowledge is distributed (translated, transformed, and adapted) through participation in the exchanges of concrete professional communities, guarded through social transactions and relationships. Such process is rooted in material contexts and, therefore, conveyed through objects and artifacts; it is also reflective in that it is exposed to the accountability dimensions relating to one’s social recognition and reproducibility (see Scaratti and Ripamonti, 2015).

The acknowledgment of situated and practical dimensions of knowledge is found in the evolution of theories of knowledge in the 20th century that led to the progressive overcoming of the positivist and neo-positivist perspective with the acquisition of the basic assumption about the non-neutrality of the empirical data. Theoretical points of view, pre-understandings, and perspectives guide the processes of interpretation/attribution of meaning and construction of data, which are precisely given (offered) and constructed at the same time. The plausibility of their knowledge depends on an intersubjective validation to be made explicit and regular, for which the production and communication of knowledge seem irreducibly entrusted to a triangulation between empirical references, languages, and discursive practices in use, and theoretical-conceptual frameworks adopted. A work of constant attention and regulation of the implicit elements at play (in the tension to make them explicit and specify them critically) is needed; however, this does not imply adhering to the extremes of skeptical drifts of a postmodernist and de-constructivist mold. The image of the platypus, an animal that with its surprising strangeness defies any classification and the very possibility of thinking and representing it (see Eco, 1997), becomes a metaphor for indicality or indexicality (Peirce, 1955): a relationship of real connection or dynamic coexistence between index sign and object to which it belongs. It involves the possibility and conditions of recognition of things (known and less known) and the semiosis present in perceptual processes, for which when we are surprised by something, we take hold of facts, issues, and objects, or we experience something that takes us. The indexical forms of communication (such as pronouns and deictics in language) require their interpretation of the situation in which they are expressed, so implicitly in a given context, we recognize that a question is a question expected to be answered, that a discursive practice is an accusation to which a defense is connected, or that a gesture is a request for courtesy that produces a sign of availability or a justification for any denial.

It is within such a practical sharing of meanings (Schatzki et al., 2001) that contexts take shape with relational and material dimensions and knowledge is configured as a situated activity in which gestures, esthetics, methods of interaction, organizational rules, technologies, and discursive practices are combined. Referring to this knowledge (similarly to the multiplicity of components and forms involved in the case of reference and understanding of the platypus) mobilizes an articulated plurality of attentions and devices, requiring each time to move between observations and field notes, analysis of artifacts in use, transcripts of discursive practices, and reports and tales from the field (Van Maneen, 1988).

The relevance of work contexts as particular and elective fields of research and loci of the multiple variables and implications of work processes reconfigures the relationship between researcher and research object; there is a need for a reconsideration of the connections between approaches and disciplines of knowledge (such as WOP) and the possibility of producing knowledge starting from problems generated by organizational contexts and in function of a possible change.

### The Emic and Situated Methodological Approach

Dealing with situated knowledge entails important *methodological implications*, with privileged attention to fieldwork and a view on proximity to operational practices, through methodological approaches that are functional (Charreire-Petit and Huault, 2008) to grasp elements of singularity, detect meanings, cultures, interpretations, and attributions of meaning to events and actions. The knowledge generated and acquired in this way seems to facilitate the access to possible windows of understanding on working and organizational culture; on circulating criteria of utility (what is needed and not needed) and judgment (what is considered adequate and inadequate, appropriate and inappropriate, and right and wrong); on representations, readings, and ways of seeing and thinking; and on routines and meanings more or less shared by subjects about their own reality and organization.

The most congruent methodology for the investigation of the meanings built around objects moves between “ethical” approaches (attentive to the comparison and transferability of knowledge) and “emic” approaches (linked to the possibility of accessing discursive, conversational, and narrative universes, to artifacts and reports of work practices) as an empirical expression of the voices of organizational actors who negotiate, reinterpret, modify, and reconfigure their rational knowledge (Pike, 1967; Berry, 1969, 1999).

Gherardi (2006) suggests to identify as a unit of analysis the processes through which practical knowledge becomes institutionalized and in which knowledge in practice becomes more accessible and detectable: the arrival of a “novice” into a context of practitioners (referring to the interweaving of experience and competence described by Wenger, 1998); the processes of circulation and exchange of practices between interdependent communities (related to the management of border relations and the translation of practices into discursive and implicit accounts, which makes them understandable in a context); situations of rupture and “repair” of the practices in use (connected to the dynamics described between reification and participation); and network situations at an inter-organizational level (recalling the role played by speeches, brokers, facilitators, and intermediaries of various kinds). The possibility to intercept and approach these situations allows one to describe the process of reproduction of practices over time, capturing the aspects of dissemination (whereby some practices are replicated by incorporating differences) and discontinuity.

Silverman (2007) also suggests a series of indications that connect the recognition of situated knowledge to an elective approach of qualitative research, able to ask questions about everyday routines and to pursue a reasonable regulation of the investigation devices, according to a logic of theory of the technique that connects object under study, theoretical paradigm, and situated context. The author distances himself from a psychologistic and individualistic conception of access to knowledge, to highlight the more markedly and structurally social aspects of “what happens between our ears.” It is crucial to observe how people make publicly available, in countless life contexts, experiences, and reasons connected to them. This means discovering the social order in the tiny social construction activities of everyday life, describing the categories used in social life, and focusing attention on what people do and how they do what they do. The research object is thus configured as an investigable set of describable practices and the contexts that the subjects ordinarily constitute for themselves become the default source of the data. Getting close to the accounts produced by the expert system (of their own practices), in a perspective of co-construction of the corpus of data, clarifies and specifies the relationship between observation and categories/pre-understandings inherent to the research questions identified.

The possibility to observe a set of activities led by people faces us with encountering the details of social action in a rigorous, empirical, and formal manner, in ways other (although not opposite) than a psychological tradition related exclusively to experimental and statistical methods: The author advocates for a practical ethnography, whereby the interpretations of the subjects are not limitless or purely formal, as the practitioners of daily life not only interpret their worlds, but they do it based on expectations and according to recognizable agendas. The strength of this approach consists precisely in studying phenomena that are simply otherwise not available: their locally constituted configuration in specific contexts and situations.

Silverman’s contribution highlights how social realities are constantly under construction, through sense-making processes starting from practical issues. The context itself is not something defined once for all; it is shaped through the concrete interactions of people in real time.

The reference to the action of subjects in their contexts mobilizes a conception of knowledge different from only existing in people’s heads and attributable to storable and transferable information but as structurally connected to systems of activity (Engeström, 1987) within which it emerges as the result of daily connections between subjects, relationships, organizational and institutional variables, artifacts, material, discursive, and social mediators, according to forms in constant evolution.

### Embracing a Relational Practice Approach

The option of a mediated realism, the assumption of a practice-embedded knowledge, and the adoption of an emic, situated, and transformative methodological approach introduce the theme of processuality and relatedness connected to them.

Producing knowledge in/for contexts involves the possibility (not taken for granted and to be legitimized) to enter them, exercising and modulating one’s power of influence, orientation, negotiation, and promotion of interests and agreements to enhance growth processes, starting from problems, needs, and solicitations identified and agreed. Languages, symbols, and identity dimensions can be understood within a system of networks and social relations of which the various organizational actors constitute as many nodes. Such knots are shaped through processes of belonging and interaction also in the light of an evaluation of opportunities and the risks of the transactions in which they are involved (Granovetter, 1985).

In this perspective, studying organizational realities, intervening in them, and generating knowledge and changes mean approaching contexts of action characterized by uniqueness, ambiguity, unpredictability, provisionality, and measuring oneself with practical and implicit knowledge.

Hence, we encounter the challenge of dealing with research objects that increasingly take the form of research problems, generated by situations of urgency and low formalization, the configuration of which derives from social exchanges with differently experienced interlocutors. Such objects depend not only on the logical coherence of the project that connects them to a theoretical paradigm of reference but also from the process of concrete construction linking the problems to knowledge objectives that can be spent and consensually recognized.

To produce such kind of knowledge, the researcher needs to *construct a relation* with the context and its actors based on mutual trust and not built in a day. It requires familiarity with the reality at hands and entails times, reciprocal acknowledgement, and hyphen-space balance (Cunliffe and Karunanayake, 2013) between all the co-researchers involved, seeking to respect the particularity related to each case and situated context of research. Since the inquiry process usually takes place on the field, inside the systems of action, the objects of the research are constructed by the negotiation and dialogue between researcher and stakeholders, enhancing a good enough reciprocal position and engagement (Cunliffe and Scaratti, 2017).

We can highlight some relevant features that characterize the *relational stance* of research, due to the requested modulations and declination related to the engagement degree of the researcher in the field, the configuration of the objects, the negotiation of time and resources investment, and the identification of the different expectations about the process, methodology, and products of the intervention. At stake are different levels of relationship:

- the relation between theoretical pre-comprehensions that guide the observation on the field and the unfolding process of grasping reality through gazing and glancing processes, dealing with the need to detect multiple points of view, representations, and readings of the various organizational actors concerning the organizational action they are part of and which they contribute to determine;
- the relation between researcher and other stakeholder involved, according to the perspective of knowing from within (Shotter, 2008, 2010) and the need to achieve a good enough distance. The metaphor of the hyphen-space (Cunliffe and Karunanayake, 2013) symbolizes the right position and the boundaries set in place between the various actors involved in a research study: they are not taken for granted and have to be achieved among four principal areas of relational tuning: internal-external, similar-different, involved-distant, and politically active-active neutrality role, in relation to the exercise of own power and influence;
- the relation between the particular and general, since particular cases without general concepts are blind, while general concepts without particular cases and accounts are lame;
- the relation between academic and practitioner interests, due to the need for combining those into a production of knowledge both relevant and actionable for the professionals and suitable to be spent for scientific communities.

Relational research faces complex weaving of explicit and implicit dimensions, aspects embedded and distributed in social practices, tacit knowledge whose detection enables one to intercept how the local reality is constructed, what processes it generates, if and how it can be changed. All participants are called into question in accordance with appropriate regulations to be agreed upon, as negotiators, co-authors and players of events, practices, and comments to be included in the accounts (Cunliffe, 2003). The challenge is the possibility of acquiring different types of accounts (discursive, conversational, and narrative; artifacts and reports of practice) as an expression of the voices of the organizational actors who negotiate, reinterpret, modify, and reconfigure their practical knowledge: such a possibility relies on a dialogical, situated and unfolding negotiation of plural issues at hand, that is on relational practice.

Taking into account different voices and enabling the effective and active participation of people at all stages of the process of knowledge production, especially of those who have direct experience of the subject matter investigated, requires a careful recognition and verification of actual conditions of practicality and sustainability for such an approach. On one side, it demands an explicit agreement between the practitioners involved; on the other side, it asks for an institutional alliance that legitimates the investment of (tangible and intangible) energies and transaction costs related thereto. The willingness of participants in the co-production of materials, texts, documents, stories, accounts of various kinds must not be taken for granted; this includes the activation of devices and situations capable of generating and sustaining processes in which the different subjects can meet, review gathered practices and knowledge, and negotiate meanings. The complexity of organizational life suggests the implementation of a set of dimensions of care, support, and listening that must guide the assumption of a task starting from the real problems identified, deciding opportunities, methods and levels, variations, and conditions of use.

## Walk the Talk: a Relational Research in Practice

To focus on the translation of the theoretical framework depicted above in a concrete research experience, inspired by such a WOP applied orientation, we touch upon a brief exploration of a case study related to a research project commissioned by the national board of an important Italian voluntary blood donors organization. The utterance “walk the talk,” assigned to the research in the moment of its conclusion, emblematically synthetizes the peril of a gap between the declared values and the daily practiced experience, reminding the need for coherence and responsibility rooted in practice evoking the separation among general and particular, theory and practice.

While we refer you to another paper (Cunliffe and Ivaldi, 2020) regarding this case for a detailed analysis of the concept of embedding ethics in specific situations and the discussion of the findings acquired, in this contribution, we address the most relevant steps of the research, concerning the relational dimensions at stake. After a brief description of the context, we point out some key aspects in which research as a relational practice is highly solicited. We seek to point out the research process, providing situated support to the theoretical framework, as an emblematic practical repertoire of the conceptual background adopted, suitable to be transferred in different and plural contexts.

The research took place in an Italian blood donors association acknowledged by public institutions and laws as a private and not-for-profit organization with the public aim of assuring a sufficient supply of blood and its products for every need of the patients, with a voluntary and not remunerated donation. Founded at the beginning of the 19th century, its history has developed and consolidated up to the current configuration, counting about 1,320,000 associate members, a national headquarters and 3,200 local sites distributed at the provincial (120) and regional (22) level, each with its local institutional government to manage the system of activity of blood donation.

The associates have different roles and different types of commitment toward the organization: among the volunteer donors, there are some members (democratically elected for each local, provincial, and regional site) that have a managerial role with the task to define the strategies and administrative issues of the organization. Along with that, some of the local sites (those that directly collect blood) commit part of the activities – desk and medical work – to personnel that is employed. The incremental growth of the association has brought to a high complexity and articulation of the organizational processes, triggering a diffuse feeling among the associates: They feared that the organization could risk losing a consolidate identity, its related objectives, and mission generally shared by the organizational players. The possibility to work throughout the country, thanks to the dislocation of the local sites, constitutes both a source of opportunity and a source of fragmentation and dispersion. It constitutes an opportunity because it generates a social impact on the territory and, therefore, major changes on the national country; it also facilitates fragmentation because it can bring organizational players to work and operate in a dystonic way with respect to association objectives and mission. In this sense, the threat is that of a loss of the organizational identity, a lack of shared objectives and goals and a widespread pursuit of individual interests that could be in contrast with the creation of the common good for society.

This perceived risk of fragmentation brought the association national president to ask the authors of this paper for a consulting intervention seeking to write a code of ethics: A list of values and prescriptions that could help the organizational players in their everyday behaviors and practices.

Despite the brevity of the description of the context, it is enough to evoke the complexity of the dimensions involved and the need to deal with the multivocality, process-oriented, open to the transformative lens features that underpin the adoption of an applied approach in such a contest. Researchers have to face institutional, organizational, relational, and practical issues, related to different and plural embedded cultures, habits, and practices, that constitute the idiographic stance of the particular landscape at hand. The demand for a code of ethics opens a possible interpretation as an opportunity, a portal for new and unexplored ways of thinking and seeing about ethics, promoting different and innovative forms of understanding and interpreting it, acknowledging the existing criticalities and problems, dealing with their sustainable transformation.

The applicative stance of WOP here matches the singularity (the particular) of the organizational context, including its complexity and the challenge to weave and develop a texture of relationships among the plural levels at stake, starting from the trust conveyed by the request addressed to the authors of this paper. Adopting a logic of practice, we coped with the need to share both a more detailed definition of the problem and the research questions, moving from concrete, lived issues and the suitable and sustainable ways to produce knowledge, pointing out the situated experiences of the subjects as the constitutive and privileged ground from which gathering relevant knowledge.

In the following paragraphs, we describe the exchanges, transactions, and negotiations between researchers and subjects that underpinned the research process in its unfolding evolution.

### Reshaping the Object of Research

A first relational step of the research emerged at the beginning: As researchers invited due to the trust acquired in previous training interventions with the board of the association, we were faced with the condition to both accept the request (helping to write an ethical code) and negotiate it, reshaping the object through a dialogue between our theoretical pre-comprehensions and the multiple expectations and desires of the organizational stakeholders.

The relational step related to the object was about the dialogue among two perspectives. On one hand, there is the implicit assumption of a deterministic approach considering ethics as grounded in universal laws and principles that can be applied to every organizational context without any difference: The ethical codes become a collection of universal and theoretical values that, in the representation of the managers, can influence and determine the behaviors of the players. On the other hand, we have a perspective that sees ethics as a strongly contextual and situational aspect, open to different interpretations and processes based on self and group interests: the ethical codes become an ethical chart (Cunliffe and Ivaldi, 2020) depicting situations and events that open the possibility of seeing and reflecting on what people do and of doing actions that are reasonable for all the organizational participants, achieving social and organizational practices actionable in the wider organizational context.

The result of such a negotiation was the agreement to address the real life and practices of the associates, exploring experiences of ambiguity related to ethical dimensions and suggesting the creation of moments and settings in which people reflect, interact, and negotiate meanings (Shotter, 1993), collectively constructing new perspectives and actions. The object became the promotion of an organizational ethical culture inside the association, detecting ethical contradictions and spreading processes of discussion and negotiation about the meanings attributed to events and situations, roles and visions, as well as problems. It was a work of relationship-building and mutual positioning, which underlines the difference between stakeholders involved and their different interpretations and use of ethical values/principles. The first key point of a suitable repertoire transferable to other contexts is shaping and defining the object of research as a socio-constructivist process among the plural stakeholders involved.

### Achieving Good Enough Conditions for a Knowing From Within Practice

The challenge to improve a commonly acknowledged ethical culture faced the need to promote the involvement of professionals and organizational stakeholders at the various organizational levels (Engeström, 2005). In this way, it is possible to improve real experiences of the organizational participants, dealing with the interplay between the internal organizational context, managerial interventions, the institutional and normative environment, and the complexity and uncertainty of the social and material processes at stake, following two relational directions.

The first trajectory concerns the need to activate a *temporary organization* able to set up adequate social space-time moments, as well as to mobilize concrete modes of work (producing documents and collecting material), reciprocal role attributions (dividing tasks and exchanging expectations), access conventional materials and technologies/tools in use (proximity to the operational environment). A stipulation of rules and agreements is needed, as well as the shared configuration of the products expected from the research. This led to the creation of a heterogeneous advisory committee: Six representatives of the association (at different hierarchical levels) were selected to be part of this committee, together with the researchers. The underlying spirit was that of working and moving toward the creation of ethical culture and positive social change that can be more applicable to local communities by insiders who work as collaborators.

The task of the advisory committee was to participate in all the phases of the research intervention, making it possible to detect contradictions and compare interpretations between researchers and members on equal terms and with reciprocal validation. The advisory committee as a temporary organization facilitates knowledge production and its translation into real practice and system of action. Such a knowledge transfer is not linear and straightforward, since it must face with materiality (artifacts, technologies used, and dimensions of space and time), rules and agreements on social exchange (sequences, tasks, and conventions), and constraints (roles, coordination, expected results, and management methods), which characterize the organizational conditions that allow its real possibility. The relational stance of this step refers to not overlooking the mobilization of institutional, organizational, logistical, tangible, and intangible conditions that enable subjects to invest in the processes through which they can build something in context with other people, starting from common investments. Since the production of knowledge is close to the organizational processes and real problems that the subjects face, providing suitable moments in which they can revise and reorient their frames of reference, transforming practices recognized as inadequate and grow in their work experience, becomes of strong importance.

The second direction is about the promotion and creation of pivotal groups that, as communities of practices, enhanced the mutual engagement linked with the dynamics of interacting together socially; this supports a joint enterprise related to some kind of shared norms and accountability in behavior, a common repertoire related to the circulation of shared stories and concepts related to practice (Wenger, 1998).

In this framework, the circulation of knowledge and the relationship between people become fundamental and crucial to achieve a social identity and interests and expressed values (Newell et al., 2009).

Since the objective of the research became the identification of situated criticalities and problems related to ethical aspects while seeking their acknowledgement and management, dealing with the temporary organization and community of practice orientation provided the right direction and sense to be pursued. The symmetrical power and the agency were valorized by both researchers and internal stakeholders, achieving a good reciprocal position in influencing, endorsing, promoting, and sustaining the unfolding research process.

The two relational steps we described are strictly related to the applied and situated research perspective adopted since they convey a close connection among the different levels and voices involved in the complex and multifaceted environment. They are also relevant in supporting the link between searching for situated knowledge (including criticalities and contradictions) and the practitioner participation in the reflexive path that it triggers.

These relational steps developed processes of participatory research and reflection, helping to:

- negotiate the proper methods to detect the main critical aspects and the ethical problems faced by the different stakeholders;
- analyze the ethical principles and values that were socially recognized within the association;
- promote among the organization members the social and material conditions to activate changes in order to improve the association environment;
- share an ethical culture inside and outside the organization, seeking to consolidate a clear identity of the association and reduce difficulties and contradictions connected with ethical issues.

A narrative and action research approach was agreed upon and used in order to support how the participants interpreted their ethical experience. As a result, a large number of stories were collected, enabling the description of emblematic and diffuse ethical dimensions and contradictions to promote their acknowledgement and possible transformation. The collected stories and contradictions were used to create the ethical chart of practices, designed as a flexible document and thought as a practical tool useful to provide opportunities for people to question their actions and confront the critical aspects of their everyday behaviors (Cunliffe and Ivaldi, 2020).

The enhancement of a temporary organization and the promotion of communities of practice constitute a second key point of a suitable repertoire transferable to other contexts.

### Matching Academic and Practitioner Interests Through Transformative Intervention

As discussed in The Transformative and Social Accomplishment Stance of Applied Science section, a crucial and critical point in doing research as relational practice entails its transformative configuration, in other words, the possibility to use the acquired knowledge to change and develop the acknowledged criticalities. In our case study, this aspect relies on the use of the ethical chart as an artifact that can trigger and facilitate the attribution of meaning to events and problems, the development of change actions, and the enhancement of local contexts. At stake is the achievement of a strong alliance between organizational stakeholder and researcher addressing the common investment in a transformative stance. For this work of research, an agreement was made on the use of Change Laboratory approach (Engeström and Sannino, 2010; Engeström, 2011) that provides suitable conditions to promote and facilitate the transactions and negotiations between the different actors involved. Following this approach, a pilot formative intervention took place in a provincial site of the association intending to test the use of the ethical chart of practice. In this perspective, the activation of the Change Laboratory represented a turning point for the expansive learning process in the blood donors association: a shifting step from a phase of inquiry and detection of criticalities and contradictions toward the configuration of new forms of practice.

This required, from a methodological point of view, the creation of a formative intervention able to support the translation into practice of the ethical dimensions and emblematic repertoires of action identified during the research phase and collected in the ethical chart of practices. We asked people to directly compare their situated experience with the stories and the questions prompted by the ethical chart of practice. Contradictions and challenges identified during the ethnographic fieldwork were used during the Change Lab as “mirror materials” to stimulate collective analysis, problem finding, and developmental efforts by the participants. The ethical chart of practice, collecting stories that show value dimensions and critical issues, problems, and contradictions related to the organizational life, was conceived as a *second stimulus* prompted for generating the process of acknowledgement and transformation of the ethically scattered organizational conditions of the association (*first stimulus*; Sannino, 2015). We can see this use of the ethical chart as both a meaningful example of how theoretical and practical interests can be intertwined, and as strictly connected with another distinctive feature, described in The Particular as a Mirror of the Whole section, of the applied and situated research perspective adopted, related to the relationship between the particular and the whole. Needless to say, the pilot experience generated high levels of interest and participation, bringing together action, reflexivity, theory, and practice and generating hints and practical suggestion for the whole association.

Selected associate members coming from different regions participated as observers to the pilot process, seeking to achieve competences and learn the transformative approach, having received an institutional mandate to spread and replicate the experience in other local sites.

The transformative stance has a relevant implication in the relational practice of research since it entails a strong work of social texture, promotion of conversations, discussions, and negotiations, enhancing the possibility to create critical encounters among different levels and multiple players of the organizational context.

The achievement of an agreement about the investment in a transformative intervention inside the organizational context constitutes a third key point of a suitable repertoire transferable to other ones.

## Discussion

The case study of the blood donors association shows how a relational research approach can enable effective utilization and translation into action of the ethical chart of practice. The transversal empirical narratives (*general* accounts of a multifaceted organization) are useful for triggering an expansive learning process in a specific context (the *particular* situation of the pilot experience), while what is learnt inside the specific situation (the particular) can improve the whole understanding of the ethical dimensions at stake (the general).

This refers to the relationship between uniqueness and generalization and the connected implications for the ways of conceiving replicability in WOP.

Specifically, we aim to underline how the knowledge transfer generated by limited situations and replicated in a wider context requires the creation and management of complex processes of widespread learning. In this regard, Carlile (2002, 2004) establishes how the transfer of organizational knowledge involves competencies and processes of knowledge transferring, translating, and transformation.

In our case, the presence of the associate members as observers of the Change Laboratory sessions and their activation as boundary spanners, with the task of accompanying similar processes in other sites, highlights an important critical aspect. Transferring knowledge in these cases cannot be identified with replicative and standardizing mode; rather, it requires an activation of experiences through which participants relate to the problems of their community life and build hypotheses to address and change them.

From this point of view, the attention to disruptions, contradictions, and conflicts can represent, for people engaged in practical situations, an opportunity for transformative agency and expansive learning (Sannino et al., 2009; Engeström and Sannino, 2010, 2011; Sannino, 2011).

Such an approach entails the need to achieve not only a detailed description of work life but also a deeper understanding of practices and their relationships as meaning-making and order-producing activities. This represents a sort of common body of relevant knowledge that allows people to situate themselves in relation to specific requirements, rules, and constraints, shaping a socially defined and constantly refine ethic (how to behave) and esthetic (what and how to see) judgments, by which they share what has to be considered as correct or incorrect, right or wrong, and tasteful or distasteful.

At stake is the possibility to give importance to micro-cultures, the problematic aspects of the professional and relational practices of working life, and the role of reflection in shaping knowledge and professional expertise. We have to deal with a production of knowledge through an open process of discussion, active listening, and the involvement of the practitioners working within the organization (the “expert system” because they possess evaluative knowledge in regard to judgments, criteria, tacit beliefs, and assumptions). It is a relational and social process (Scaratti et al., 2017b), concerning the need for constant transaction and negotiation with the organizational players (each with their distinct representations and interpretations) in order to agree on the aims and methods of knowledge shaping.

However, the possibility to involve practitioners as co-producers of situated knowledge is not a condition to be taken for granted: It requires spending organizational, mental, and relational energies to organize working spaces and settings, acquire the materials (texts, documents, artifacts, and accounts) that the people involved are asked to produce, and use techniques and instruments that enhance the participation and collaboration of various interlocutors. We must shape a suitable climate due to the possibility that practitioners are solicited to reveal aspects of their work and features that they may perceive as unpleasant and threatening. It is, therefore, essential to create conditions that help them to describe and speak in a free and sincere way about their work and professional experience. As people are requested to become like researchers into their own professional experiences, the creation of alliances with the organizational board to facilitate negotiations and exchanges among practitioners is crucial.

According to this perspective, the opportunity to detect and understand situated knowledge, to reflect upon and transform routines and practices can be conceived as an unfolding process of accomplishment (Orlikowski, 2002) in which methods and tools are context-dependent and relentlessly subject to negotiated, ongoing, and constantly refined mobilization of knowledge.

## Conclusion

In this contribution, we have explored the epistemological and theoretical assumptions related to the adoption of WOP as an idiographic, applied, and situated science, pointing out the ontological, gnoseological, and methodological implications concerning this orientation.

The paper highlights how this kind of research approach allows the researcher to move through different levels of the socially constructed reality. Adopting such situated perspective entails aiming to produce knowledge that is meaningful and relevant for the stakeholders since the identification of problems depends on the representations of the actors and their demarcation is not immediately obvious (Alvesson and Sandberg, 2011; Sandberg and Alvesson, 2011). The methods, techniques, and tools themselves cannot be always defined in advance; rather, their choice and the adoption of the most suitable one need to be progressively negotiated during the research process.

We argue that the adoption of an epistemology of the particular and a practical rationality rests on a “weak and shrewd constructionism” that distinguishes the processes competencies in organizational and management research (how to deal with a social accomplishment effort) from the skills related to the production of the research data. It is not a matter of ideological positioning but of the professional and aware achievement of the capability to address the constant declination between the problem to be faced, the available theories, and the use of proper and coherent tools (situated demand and methodological bricolage) in a multi-paradigm perspective able to control the conditions of *teckne* (lenses, method, and tools) and the relational process at stake to produce suitable and relevant knowledge.

To this regard, Cunliffe (2011) claims the usefulness of advancing toward ever more attentive, informed, thoughtful forms of studying and theorizing about the complexity of organizational life. She proposes the adoption of a *reflexive hermeneutic* perspective (Cunliffe, 2011), which seeks to understand how we share processes of interpretation and attribution of meaning to the world by interacting with each other within a context. Three knowledge issues are suggested (objectivism, subjectivism, and intersubjectivism), highlighting the structural intertwining of subject and object within tangible, technological, symbolic, and relational transactions, seeking to figure out the question related to what (and how) something may be investigated by a social science at a given point in time. The relationship between the three *knowledge issues* is not represented in terms of different features placed in a linear sequence (objectivism-subjectivism-intersubjectivity) but as a field of complexity and tension, which supports the configuration and craft of new avenues of research, with greater meta-theoretical awareness in the study of organizations, dealing with blurred and permeable boundaries.

To sum up the main issues we stressed in our contribution, we argue that applied and situated research practices, one that is able to unleash the cognitive potential of practical knowledge and to configure relevance (for practice), can be conceived as one of the criteria for scientific legitimacy of knowledge. The structural connection attributed to the relationship between knowledge and action (Tsoukas, 1994, 2009) makes it possible to go beyond an epistemology of representation and accept the suggestions of an epistemology of practice. It sustains the ability to approach (to determine the correct distance from) experience, capturing the indexical nature of emerging practices, the elements taken for granted; it also supports the ways to build, shape, sort, and justify experience and the methods adopted to relate to others and make connections between the activities in which one is engaged. The challenge lies in the issue of practical relevance and producing knowledge both close to the problems and relevant, capable of directing realistic, significant, and sustainable solutions for the people involved. Lewin has stated that there is nothing more practical than a good theory and, in fact, there is always a need for better theories than the previous ones. However, it is the way in which a theory is used in concrete practical contexts that is important, as it is the translation made of it into practice that marks its difference. This means giving meaningfulness to local experience and situated knowledge, implicit epistemologies, and knowledge informally distributed and socially safeguarded, which takes shape within the practice of action in an organized context.

This also conveys strong relational features, since knowledge production constantly moves between aspects of investigated reality that are socially constructed (through conversational, linguistic, tangible, and symbolic processes), that stimulate the researcher to situate him/herself in a constant adjustment of position, reflecting on his/her thinking process and thought within a dialogic process already initiated (Shotter, 2010), and that must intercept processes emerging from the development of events, situations, and changes of scenarios that occur at various levels.

In a synthetic note, we claim that a good and situated dialogic transformative research is a product-process (1) collectively acknowledged as relevant, (2) effective and useful to tackle with problems, (3) close to people representations, and (4) meaningful for both the researchers and the actors.

However, we cannot avoid also limits related to our argumentation for the idiographic, applied, and situated configuration of WOP as social science. Some aspects need to be better explored:

- how to combine the need of a supervision of the empirical and methodological anchors of the data with the request of a co-generation of knowledge with practitioners;
- how to avoid the risk of a reductivist adoption of applied knowledge as instrumentally enslaved to economistic or opportunistic needs;
- how to conceive and combine the different setting of research and application.

These and other issues could constitute important stimuli for further research and studies.

As a conclusive remark, we can recall the plausibility and usability of advocating an authentic applied stance as a relevant (albeit not exclusive) lens in conceiving WOP as a social science, able to enrich and increase widespread forms of knowledge production in this discipline. Such a declination concerns the possibility to cope with problems that are generated by real contexts in which work and organizational experiences take place; it also configures a way of approaching research as *context-driven*, capable of enhancing the diversity of circumstances, the integration of different disciplines, and aspects of a socially effective applied psychology.

## Author Contributions

Both the authors discussed and wrote the paper, having shared the on field experience described in the contribution as a trigger for epistemological considerations. All authors contributed to the article and approved the submitted version.

### Conflict of Interest

The authors declare that the research was conducted in the absence of any commercial or financial relationships that could be construed as a potential conflict of interest.

## Footnotes

^1^https://pubmed.ncbi.nlm.nih.gov/31596559/
